# Sizes of atmospheric particulate matters determine the outcomes of their interactions with rainfall processes

**DOI:** 10.1038/s41598-022-22558-6

**Published:** 2022-10-19

**Authors:** Relotilwe Maboa, Kowiyou Yessoufou, Solomon Tesfamichael, Yegnanew A. Shiferaw

**Affiliations:** 1 Department of Geography, Environmental Management and Energy Studies, University of Johannesburg, Johannesburg, South Africa; 2 Department of Statistics, University of Johannesburg, Johannesburg, South Africa

**Keywords:** Environmental sciences, Atmospheric science

## Abstract

Environmental sustainability remains at risk, given the coupled trends of economic development with air pollution. The risk is even greater in the water-stressed world, given the potential suppression effects of air pollutants on rain formation. Here, since these suppression effects remain debated, we tested the hypothesis that air pollutants suppress rainfall in the water-stressed South Africa. This was done by fitting generalized linear models to a 21-year historical dataset of rainfall and air pollutants. We found that some gaseous pollutants and PM_10_ show a significant negative correlation with rainfall, perhaps due to the temperature inversion they cause, which might prevent the upward rise of humid air and convective clouds to grow high enough to produce rain. Surprisingly, as opposed to PM_10_, we found a rather positive significant effect of PM_2.5_. Altogether, our study supports the hypothesis of rain prevention by pollutants but provides some nuances that are dependent on the size of air particle matters. To achieve environmental sustainability while growing the economy, we can only rely on emission purification technologies to strike this trade-off.

## Introduction

Water scarcity—the lack of sufficient water in certain parts of the world—has plagued the twenty-first century^1,2^. Water scarcity across the globe is a threat to human health, the environment, food supplies and socio-economic development^3,4^. Around 1.2 billion people do not have access to safe drinking water in the Global South, and an additional 700 million people are expected to be displaced in the world due to water scarcity^5^. The International Water Management Institute predicted a severe water scarcity by 2025 for multiple countries in the Global South, due to increasing population growth and man-made pollution^6^. Also, approximately 4 billion people worldwide are predicted to be affected by water scarcity by 2050^7,8^, and the severity of the impacts of water scarcity may be worse than currently observed due to the exponential growth of human population.

Indeed, we have entered the Anthropocene, an epoch that started in the 1800s when man-made activities began to significantly impact the planet's climate, ecosystems and environmental resources^9^. In this epoch, the impacts of human pressures on the environment are outrunning those of natural environmental processes that have been shaping the environment over the past geological times^10^. One key feature of the Anthropocene is the unprecedented increase in the use of fossil fuels^11,12^, leading to massive and frequent air pollution that is putting human health at risk and jeopardizing the Earth system’s ability to provide clean drinking water to human^13^.

According to Rosenfeld^11^, anthropogenic air pollution may be aggravating water scarcity issues by suppressing rainfall processes^12,14,15^. For example, an early study reported a reduction in rainfall in Jiului Valley (Romania) following a period of poor air quality driven by particulate matters generated by thermoelectric power plants and coal mining fields^16^. Records of rainfall suppression in the Haifa Bay region, Israel, was attributed to air pollution, since rainfall delays are notably pronounced during the periods of massive urbanization and heavy industrial activities^17^. The region of Haifa Bay harbours major pollution sources consisting of coal-fired power plants, refineries, several industrial activities, and a dense population^17^. Furthermore, Jirak and Cotton^18^ investigated the effect of air pollution on rainfall along the Front Range of the Colorado Rocky Mountains (United States) and reached a similar conclusion. Similarly, in Australia, urban and industrial air pollution were reported to have completely suppressed precipitation from 2.5-km deep clouds^11^, whilst heavy smoke from forest fires suppress rainfall from 5-km-deep tropical clouds^19,20^. The mechanism proposed to explain the suppression of rainfall by air pollutants is that aerosols serve as small cloud condensation nuclei (CCN) that form large concentrations of small cloud droplets, which in turn prevent the collision and coalescence of droplets, without which rainfall-forming processes are disrupted^11,21,22^.

However, if the hypothesis of rainfall suppression by air pollution holds, we would expect that cloud seeding in a polluted environment would accelerate the conversion of cloud water to precipitation, and thus enhance rainfall amounts. Unfortunately, polluted areas are not generally drier, and rain enhancement by cloud seeding remains inconclusive in some geographies^23,24^. Even the study commissioned by the World Meteorological Organization and the International Union of Geodesy and Geophysics on the hypothesis was clear in its conclusion: “it is difficult to establish clear causal relationships between aerosols and precipitation and to determine the sign of the precipitation change in a climatological sense. Based on many observations and model simulations the effects of aerosols on clouds are more clearly understood (particularly in ice-free clouds); the effects on precipitation are less clear”^24^. Therefore, the hypothesis of rainfall suppression needs further investigation^12^, and the present study aims to contribute to furthering our knowledge on this front, particularly in the water-stressed world, e.g., South Africa.

South Africa is, indeed, one of the most water-stressed countries listed amongst the prominent southern African regions predicted to undergo severe water scarcity by 2025^6,25^. The annual average rainfall in South Africa is 465 mm, which is approximately half of the global annual average rainfall of 860 mm^26,27^. The Vaal Triangle region in South Africa is of environmental concern, due to alarming records of air pollutant concentrations coupled with both an increase in population growth and urbanization^28,29^. Comparable to the examples of Haifa Bay in Israel and Jiulu Valley in Romania, elevated air pollution concentrations in the Vaal Triangle are driven by man-made activities such as industrial processes, power generation, mining, fossil fuel burning, biomass burning, transportation, waste incineration, domestic fuel burning, water treatment works and agricultural practices^30,31^.

The present study aims to test the hypothesis that air pollutants prevent or reduce rainfall, using the Vaal Triangle in South Africa as the model system. This was done using firstly the full year data and secondly the rainy season data over 21 years. South Africa relies heavily on dirty energy sources, e.g., coal-power plants, to drive its economic growth, resulting in several air pollution hotspots in the country causing various public health issues.

## Results

**Figure 1:**
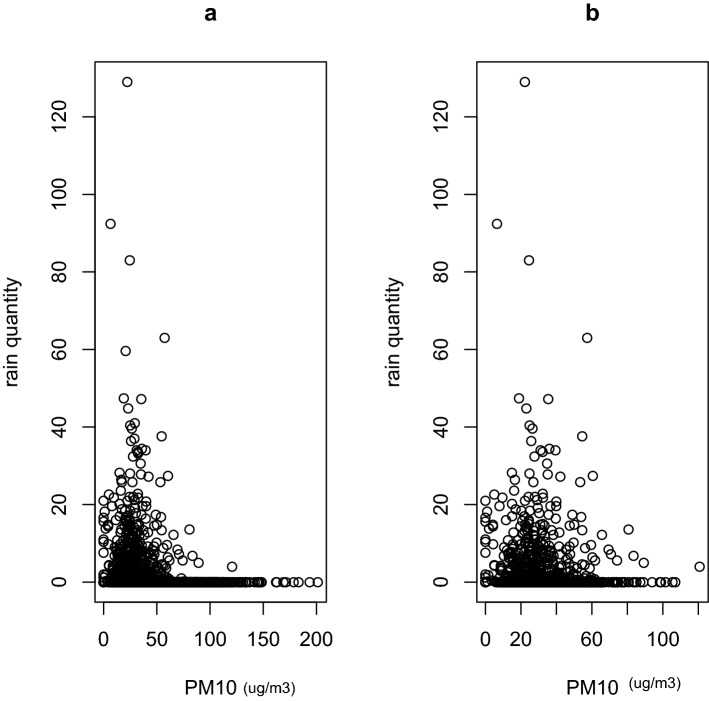
Rain quantity as a function of PM10. (**a**) For full year and (**b**) for only rainy season. Rain quantity is in mm.

**Figure 2:**
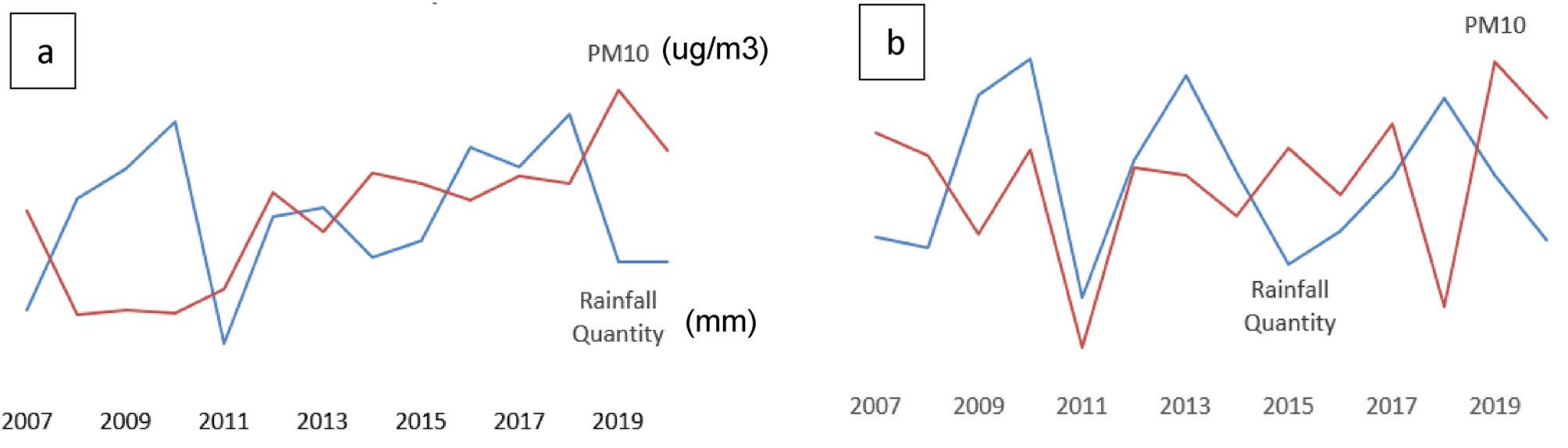
Temporal dynamic of changes in PM10 and rainfall. (**a**) Full year and (**b**) rainy season.

In terms of rain quantity, only PM_10_ among all the pollutants tested, correlates negatively and significantly with the quantity of rain (Fig. 1), irrespective of the period considered: full year (β = − 0.037 ± 0.007, *P* < 0.001; Fig. 1a; Table 1) or rainy season (β = − 0.059 ± 0.016, *P* = 0.0003; Fig. 1b; Table 2). To better understand the relationships between PM_10_ and rainfall, we reconstructed the temporal changes in both variables, and this not only confirms the negative relationships between both variables such that when rainfall quantity decreases, PM_10_ concentrations increase and vice versa, irrespective of the period cover: full year (Fig. 2a) and rainy season (Fig. 2b).

**Table 1:** Coefficients of the GLM model fitted to the full year rainfall quantity data (2000–2021).

	Estimate	Std. Error	t value	*P* value
CO	− 0.571498	0.431249	− 1.325	0.185
NO_2_	− 0.067520	0.052870	− 1.277	0.202
PM10	− 0.037150	0.007252	− 5.123	< 0.0001
O_3_	− 0.010244	0.013441	− 0.762	0.446
NO	− 0.008458	0.033366	− 0.253	0.800
NOX	0.025066	0.040241	0.623	0.533
PM2.5	0.009395	0.009188	1.023	0.307
SO_2_	− 0.034845	0.033605	− 1.037	0.300

**Table 2:** Coefficients of the GLM model fitted to the rainy season rainfall quantity data (2000–2021).

	Estimate	Std. Error	t value	*P* value
CO	0.218831	0.869746	0.252	0.801389
NO_2_	− 0.220898	0.127443	− 1.733	0.083291
PM10	− 0.059279	0.016447	− 3.604	0.000325
O_3_	− 0.013333	0.023896	− 0.558	0.576976
NO	0.001113	0.124936	0.009	0.992894
NOX	0.112287	0.116774	0.962	0.336451
PM2.5	0.022406	0.018826	1.190	0.234213
SO_2_	− 0.083906	0.060142	− 1.395	0.163225

**Figure 3:**
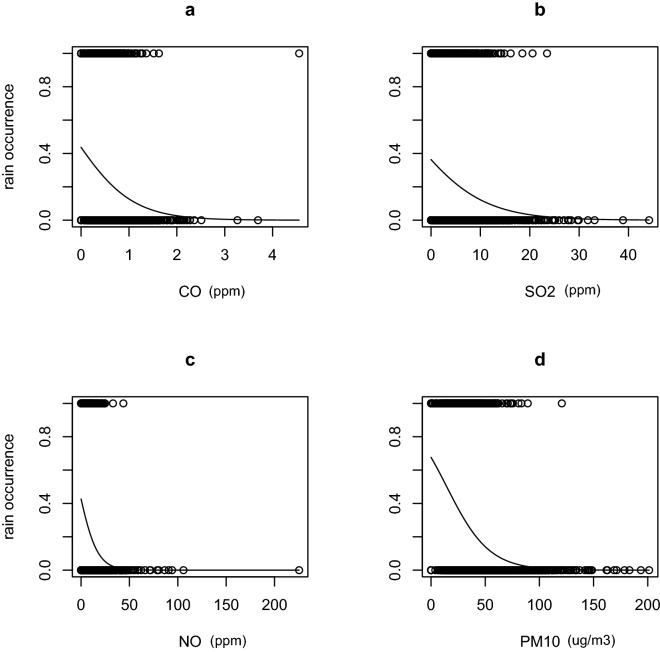
Negative relationships between certain pollutants and rain occurrence using full year dataset. Rain occurrence is measured as presence/absence.

Furthermore, in terms of rainfall occurrence, our analysis of the full-year data shows a negative effect of CO (β = − 0.713 ± 0.215, *P* = 0.0009; Fig. 3a; Table 3), SO_2_ (β = − 0.055 ± 0.017, *P* = 0.001; Fig. 3b; Table 3), NO (β = − 0.071 ± 0.031, *P* = 0.02; Fig. 3c; Table 3) and PM_10_ (β = − 0.04 ± 0.004, *P* < 0.001; Fig. 3d; Table 3). Interestingly, the effect of PM_2.5_ on rain occurrence was positive (β = 0.015 ± 0.004, *P* = 0.001; Table 3).

**Table 3:** Coefficients of the GLM model fitted to the full year rainfall occurrence data (2000–2021).

	Estimate	Std. Error	t value	*P* value
CO	− 0.713005	0.215395	− 3.310	0.000932
NO_2_	− 0.052634	0.032330	− 1.628	0.103522
PM10	− 0.048063	0.004529	− 10.612	< 0.0001
O_3_	0.003725	0.005462	0.682	0.495163
NO	− 0.071966	0.031283	− 2.300	0.021421
NOX	0.045046	0.029944	1.504	0.132496
PM2.5	0.015101	0.004800	3.146	0.001655
SO_2_	− 0.055692	0.017393	− 3.202	0.001365

Using the rainy season dataset, we confirm the negative significant effect of SO_2_ (β = − 0.072 ± 0.018, *P* = 0.0001; Table 4) and PM_10_ (β = − 0.036 ± 0.0051, *P* < 0.001; Table 4) on rain occurrence, implying that higher concentrations of SO_2_ and PM_10_ tend to correspond to the absence of rain (rain prevention). However, as opposed to PM_10_, we found a rather positive and significant relationship between PM_2.5_ and rain occurrence (β = 0.022 ± 0.005, *P* < 0.0001; Table 4), suggesting that higher concentrations of PM_2.5_ tend to drive rain occurrence (rain promotion).

**Table 4:** Coefficients of the Binomial GLM model fitted to the rainy season rainfall occurrence data (2000–2021).

	Estimate	Std. Error	t value	*P* value
CO	− 0.256753	0.244281	− 1.051	0.293233
NO_2_	− 0.058537	0.036365	− 1.610	0.107461
PM10	− 0.036670	0.005143	− 7.130	< 0.0001
O_3_	0.006655	0.006508	1.023	0.306514
NO	− 0.039687	0.035663	− 1.113	0.265783
NOX	0.055381	0.033464	1.655	0.097936
PM2.5	0.022271	0.005339	4.171	< 0.0001
SO_2_	− 0.072155	0.018901	− 3.817	0.000135

Nonetheless, the positive significant correlation between rainfall and PM_2.5_, irrespective of whether full-year or rainy season data was used, was not found in univariate models (involving only PM_2.5_ as predictor) but in multi-variate models (involving PM_2.5_ and other pollutants). For example, with rainy season dataset, we found no significant effect of PM_2.5_ on rain quantity (β = − 0.018 ± 0.016, *P* = 0.251) or on rain occurrence (β = − 0.0003 ± 0.0009, *P* = 0.735), suggesting that the positive effect reported above for PM_2.5_ is mediated by other pollutants.

## Discussion

Our key objective in the present study was to test the hypothesis that air pollution suppresses rainfall forming processes. Overall, we found support for this hypothesis but with some nuances. Specifically, some pollutants, e.g., PM_10_, show a parallel dynamic relationship with rainfall such that the increase in PM_10_ corresponds to the decrease of rainfall and vice versa. Further pollutants, essentially three colourless gases (CO, SO2, NO) also show negative correlation with rainfall (quantity or occurrence). How could these negative relationships be explained?

Air pollutants or aerosols, particularly particulate matters (here PM_10_), play the role of cloud condensation nuclei (CCN) which attract cloud droplets by adsorption. Then, adsorbed droplets may collide and coalesce to form larger drops that are big enough to fall as rain. It is worth mentioning that cloud droplets form when humid air rises and becomes supersaturated with liquid water. Then, water vapour condenses onto surfaces provided by CCN aerosols^32^. Aerosol particles, acting as CCN, lead to numerous smaller cloud droplets in areas of elevated air pollution concentration^11,22,33–35^. The smaller droplet size then suppresses precipitation formation due to a reduction of the droplet collision–coalescence process, which leads to a longer cloud lifetime as well as reduction and delay in precipitation occurrence^35^.

If the collision and coalescence of droplets are prevented (e.g., due to temperature inversion), then there wouldn’t be rain (rain prevention) or instead of being prevented, if they rather occur rarely or infrequently, there might be rain but in small quantity (rain reduction), thus leading to the negative relationships we found between PM_10_ concentrations and rainfall amount/occurrence^11,21,22^. Such negative relationships have been reported in some early studies which invoked a suppression effect of air pollutants on rainfall^11,12,14,15^ in various parts of the world, including Romania^16^, Israel^17^, the US^18^ and Australia^11,19,20^. More recently, Barthlott et al.^35^ investigated the importance of aerosols and cloud droplet size distribution for convective clouds and precipitation in Germany. The study found a significant decrease in precipitation with increasing aerosol load, due to suppression of the warm-rain formation processes^34,35^.

Interestingly, our study revealed a parallel temporal dynamic between PM_10_ and rainfall such that an increase in PM_10_ corresponds to a decrease in rainfall and when the rainfall increases, there is a concomitant fall in PM_10_ concentrations (rainfall scavenging). This coupling dynamic of both pollutants and rainfall could only be explained by firstly the beneficial effects of PM_10_ on rain formation processes as explained above, i.e., adsorption of cloud droplets on CCN (here PM_10_), collision and coalescence of droplets causing rainfall, and secondly the negative scavenging effects of rainfall wiping out PM_10_ from the atmosphere, thus causing the fall of atmospheric PM_10_ concentrations concomitantly to the increase of rainfall. Various studies have showed that atmospheric pollutants concentrations can be naturally controlled by precipitation through the process of wet removal from the atmosphere^36–38^. Specifically, Zhou et al.^38^ showed a size-dependency of the effectiveness of the removal of aerosol particles by rainfall. They showed that it is relatively easier for the rain to remove particles of sizes 2.5–10 μm from the atmosphere while those of 0.2–2 μm were more difficult to be removed^38^. They also indicated that the efficiency of aerosol removal was significantly affected by precipitation intensity such that short-term heavy precipitation helped remove particles of diameter < 2.2 μm, but long-term weak precipitation facilitated the removal of particles > 2.2 μm. The complexity of the removal effectiveness is further confirmed in a recent study which showed that the removal of aerosol particles by rainfall is influenced by meteorological conditions, raindrop diameter, and aerosol particle size^38^.

Similar to our finding for PM_10_, we also found that gaseous pollutants (CO, SO2, NO) showed negative effects on rainfall (quantity and occurrence). We put forward two possible explanations. First is temperature inversion, which is characterised by a layer of cool air in the troposphere being overlain by a layer of warmer air (the opposite characterises the normal conditions). We suggest that pollutants, including gaseous pollutants, cause the temperature increase of the air layer covering underneath the cool air in the troposphere (temperature inversion). As a support for this is a recent study that demonstrated that when PM_10_ is low, rainfall is above normal and temperature inversion is less intense than normal whereas high PM_10_ corresponds to below-normal rainfall and stronger than normal temperature inversion^39^. These findings suggest that pollutants, not only decrease rainfall but they also cause intense temperature inversion. We further suggest that this abnormality, i.e., the inversion, may act as a cap or barrier preventing the upward movement of the air as well as the diffusion of particle matters. In so doing, temperature inversion prevents convective clouds to grow high enough to produce rain, thus justifying the negative effects we found for gaseous pollutants on rainfall. Secondly, gaseous pollutants can naturally be converted into airborne particle matters through nucleation and condensation^40–43^ and this gas-to-particle conversion can be facilitated by atmospheric base species such as ammonia (NH_3_) especially at lower gas-phase acid concentrations^43^. The conversion of gas to particle therefore increases the amount of particle matters in the air, which may, following the same processes presented above for PM_10_, lead to rain prevention.

The negative effects of gaseous pollutants on rain were also reported elsewhere, e.g., India, where Shukla et al.^44^ reported that SO_2_ and NO_x,_ from various households and industrial activities decrease or prevent rainfall. Interestingly, the removal of gaseous pollutants from the atmosphere can occur naturally when the atmospheric gases are absorbed and particulate matters are trapped in rain droplets falling on the ground (i.e., rainfall scavenging)^44–48^. Again, the efficiency of this rainfall scavenging process depends on the rain droplet size and rainfall intensity, thus the relationship between the rainfall and gaseous pollutants may vary depending on the properties of the precipitation and pollutants in the atmosphere^49^.

Surprisingly, we also found that, as opposed to PM_10_, PM_2.5_ correlates positively with rainfall, but this positive effect is only found when PM_2.5_ is included in a multi-variate (rather than univariate) model where other pollutants are added. This suggests that the positive effect of PM_2.5_ is aided by other pollutants. Other studies also reported that PM_2.5_ effects are mediated by other pollutants: a reduction in CO_2_ by 10,000 t results in a reduction in PM_2.5_ by 3.3 t^50^. How is the aided effect of PM_2.5_ possible? Zhou et al.^38^ showed that it is easier for rain to scavenge particles of sizes 2.5–10 μm from the atmosphere than those of 0.2–2 μm (except in case of heavy rain; see also^37^). This means that PM_2.5_ persists longer in the air than PM_10_ when rains fall. As such, while PM_10_ have been scavenged by precipitation and PM_2.5_ persists, the collision-coalescence process of droplets^35^ around PM_2.5_ becomes more efficient, leading to rain formation, and thus the positive effect of PM_2.5_ observed in our study.

Overall, our study confirms the hypothesis that pollutants may prevent or reduce rainfall. What appears to be the contribution of our study is our finding that PM_2.5_ may promote rainfall instead of preventing it, but this positive effect may be aided by other pollutants. Our study therefore reveals the ground for cloud seedings to provoke rainfall. However, poor air quality in our study area, driven by dust and gaseous pollutants generated by thermoelectric power plants, coal mining fields and other industrial activities, is a public health concern that needs to be addressed. On this front, several environmental management initiatives have been undertaken by the South Africa’s Department of Environmental Affairs since 2006, including identification of industries causing concerning levels of atmospheric emission, setting of atmospheric emissions limits and standard, improved air quality monitoring programs, and awareness workshops promoting effective implementation of the Air Quality Management Plan^29,51^. Unfortunately, there is still no compliance that meets the acceptable standard of emissions^29^. This is not surprising given the need for economic growth which, unfortunately, relies on coal-based dirty energy that increases pollution. For example, a recent study reported a decrease in Gross Domestic Product (GDP) following a decrease in PM_2.5_ in China^52^. While we call for a ‘just transition’ towards clean energy across the globe, we highlight that in the context of urgent need for economic development which calls for industrialisation in the global south, we can only rely on technology progress, e.g., emission purification technologies, if we are to strike a trade-off between economic growth and environmental sustainability (see also^52,53^).

## Methodology

### Study area

**Figure 4:**
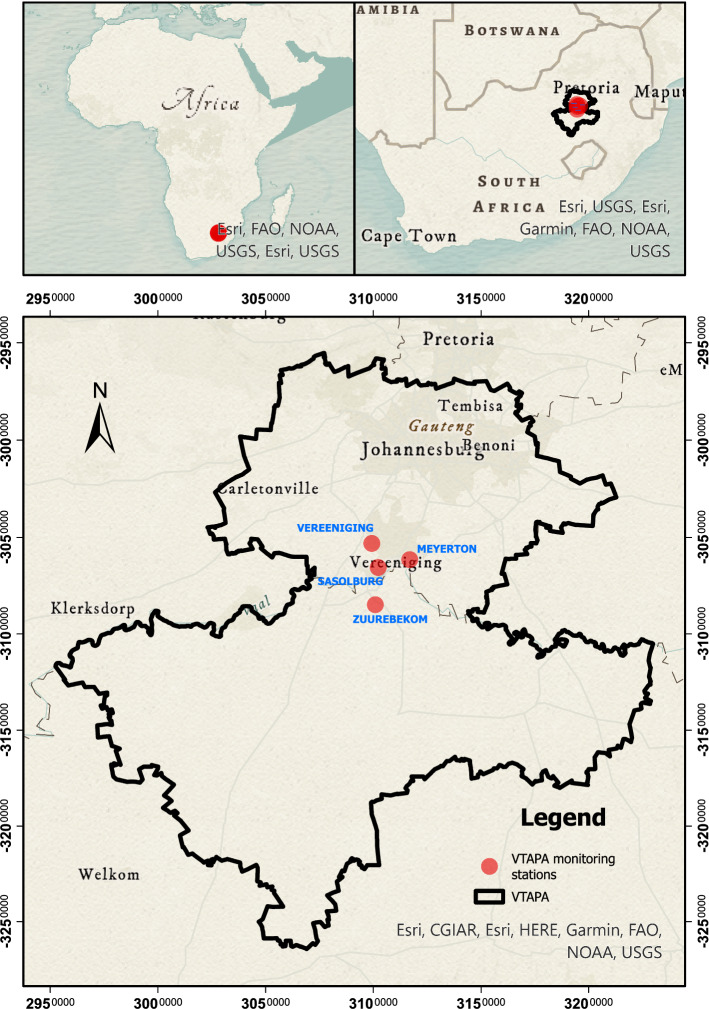
Geographic location of the study area (Vaal Triangle region) in South Africa. Map produced using ArcGIS Pro 2.9 (https://pro.arcgis.com/en/pro-app/2.9/get-started/get-started.htm).

South Africa is a water-stressed country with an annual rainfall representing half of the world’s average. South Africa shares this water stress status with several other countries in various continents around the world as well as its dirty energy based economic development pathways^54,55^, resulting various air pollution hotspots^30^. One of these hotspots, the Vaal Triangle region (Fig. 4), covers a region of over 4900 km^2^ located 60 kms south of the city of Johannesburg, stretching from Randvaal to Sasolburg in the southwest^56^. It is an industrial region plagued by persistently high concentrations of air pollutants comprising major areas such as Soweto, Johannesburg, Lenasia, Ennerdale, Orange Farm, Evaton, Sebokeng, Vereeniging and Meyerton^29^. The population of the Vaal Triangle is 3,127,907^56^. On average, the annual rainfall ranges from 671 mm in Vereeniging to 751 mm in Johannesburg. The promulgation of the National Environmental Management Air Quality Act (NEM:AQA) in South Africa resulted in the declaration, in 2006, of the Vaal Triangle as the Vaal Triangle Priority Area (VATPA), i.e., a pollution hotspot due to alarming levels of atmospheric emissions (i.e., SO_2_, benzene and particulate matters) from industrial sources such as chemical industries, petrol refiners, fertiliser production and coal mining operations^29,57^.

### Data collection

Historical rainfall amount and ambient air pollutant concentrations for the VATPA were collected from 2000 to 2021. Specifically, data on daily rainfall totals (quantity) were collected by the South African Weather Service (https://www.weathersa.co.za/home/aboutclimateatsaws, accessed August 2020) in four air pollution monitoring stations in the VATPA, including Meyerton, Sasolburg, Vereeniging and Zuurebekom. We converted these daily data into monthly rainfall quantity over a 21-year period (2000–2021). In addition, we coded monthly rainfall quantity as 1 when rainfall quantity > 0 and 0 when rainfall quantity = 0: this coded data is referred to as ‘rainfall occurrence’ data. In each of the 21-year period covered by our data, both rainfall data (quantity and occurrence) were considered firstly across the full year (i.e., Jan-Dec.) and secondly for only the rainy season (i.e., October–April).

Furthermore, we retrieved data on daily average concentrations of ambient air pollutants from the database of the South African Air Quality Information Systems (SAAQIS) (https://saaqis.environment.gov.za/, accessed June 2021) for the same monitoring stations of the VATPA. For a year, the monthly air pollution data were also considered both across the full year and also across only the rainy season. The pollutants concerned in the present study are SO_2_, NO, NO_X_, NO_2_ CO, O_3_, PM_10_ and PM_2.5_.

All data collected per monitoring station were combined to represent the data for the entire VATPA region. These data are available online at https://figshare.com/s/571736de2f84f4be615e.

### Data analysis

To test the hypothesis that pollutants prevent or reduce rainfall, we fitted the GLM model to the data collected, using rainfall as the response variable and pollutants as the predictors. Rainfall was measured in two ways: quantity of rain (continuous data) and rain occurrence defined as a binary variable coded 1 (there is rain) versus 0 (no rain). Therefore, we fitted gamma GLM and binomial GLM for rainfall quantity and occurrence data, respectively.

### The gamma regression model

Our rainfall data is positively skewed; the gamma distribution fits well with this type of data. We can model the relationship between the positively skewed response variable (that is, the quantity of rain) and potential explanatory variables (pollutants) using the gamma regression model^58^ explained below.

For y that follows a gamma distribution, the probability density function is given as:

1 $$f\left( {y; \mu , \nu } \right) = \frac{1}{\Gamma \left( \nu \right)}\left( {\frac{\nu }{\mu }} \right)^{\nu } y^{\nu - 1} e^{{ - \frac{\nu }{\mu }y}} ,\quad y > 0$$

where parameter ν > 0 is the shape parameter and µ > 0 is the scale parameter. To estimate model parameters, we used the maximum likelihood estimation method with the log-likelihood function as follows:

2 $$\ell \left( {{\text{y}};\upmu ,{\upnu }} \right) = - \log\Gamma \left( {\upnu } \right) + {\upnu }\log {\upnu } + \left( {{\upnu } - 1} \right)\log {\text{y}} - {\upnu }\log\upmu - \frac{{\upnu }}{\upmu }{\text{y}}$$

where the mean function of $y_{i}$ for the gamma regression with log link function is E$\left( {Y_{i} } \right) = \mu_{i} = \theta_{i} = e^{{x_{i}^{^{\prime}} \beta }}$ with $x = \left( {x_{i1} ,x_{i2} , \ldots , x_{ip} } \right)^{^{\prime}} , i = 1, \ldots , n$, where *p* is the number of pollutants, n is the sample size and $\beta = \left( {\beta_{0} ,\beta_{1} , \ldots , \beta_{p} } \right)$ is the regression coefficient.

### The logistic regression models

Here, the dependent variable y is binary, taking value 1 if it possesses some attribute and 0 otherwise. Since rain occurrence was defined as a binary variable coded as 1 (rain) versus 0 (no rain), the logistic regression model is ideal for modelling the relationship between the binary response variable (i.e., rain occurrence) and pollutants.

Suppose y has the binomial distribution with probability density function:

3 $$f\left( y \right) = \left( {\begin{array}{*{20}c} n \\ y \\ \end{array} } \right)\pi^{y} \left( {1 - \pi } \right)^{n - y} , \quad y = 0, 1, 2, \ldots ,n.$$

Consider the response variable ($y_{i}$; i = 1, …, n) that follows a known probability distribution that depends on a parameter θ. If we can write the function in the following form (Eq. 4), it belongs to the exponential family:

4 $$f\left( {y; \theta } \right) = exp\left\{ {a\left( y \right)b\left( \theta \right) + c\left( \theta \right) + d\left( y \right)} \right\},$$

where $b\left( \theta \right)$ represents the link function which is also called the natural parameter of the distribution; $a\left( y \right) = y$; $c\left( \theta \right)$ is the cumulant, and $d\left( y \right)$ refers to the normalization term.

The binomial distribution given in Eq. (3) can be written in the general exponential form (4) as follows:

5 $$f\left( {y;\pi } \right) = exp\left\{ {y \,ln\left( {\frac{\pi }{1 - \pi }} \right) + n\ln \left( {1 - \pi } \right) + \ln \left( {\begin{array}{*{20}c} n \\ y \\ \end{array} } \right)} \right\}$$

where the coefficient of y is the canonical parameter which is the logit of $\pi$ as shown below: $b\left( \pi \right) = ln\left( {\frac{\pi }{1 - \pi }} \right) = logit \left( \pi \right)$.

In this case, the probability of the rain occurrence is dependent on the series of pollutants:

6 $${\text{log}}\left( {\frac{{{\uppi }\left( {\text{x}} \right)}}{{1 - {\uppi }\left( {\text{x}} \right)}}} \right) = {\upbeta }_{0} + {\upbeta }_{1} {\text{x}}_{1} + {\upbeta }_{2} {\text{x}}_{2} + \cdots { } + {\upbeta }_{{\text{p}}} {\text{x}}_{{\text{p}}}$$

where $\pi$ is the probability of rain, $\beta_{0} ,\beta_{1} , \ldots , \beta_{p}$ are the $\beta$ coefficients for each pollutant $x_{1} ,x_{2} , \cdots , x_{p}$.

The probability y (rain occurrence) will take value 1 (rain) is

7 $$P\left( {y = 1} \right) = \pi \left( x \right) = \frac{{e^{{\beta_{0} + \beta_{1} x_{1} + \beta_{2} x_{2} + \cdots + \beta_{p} x_{p} }} }}{{1 + e^{{\beta_{0} + \beta_{1} x_{1} + \beta_{2} x_{2} + \cdots + \beta_{p} x_{p} }} }}.$$

The probability y (rain occurrence) will take value zero (no rain) is

8 $$P\left( {y = 0} \right) = 1 - \pi \left( x \right) = \frac{1}{{1 + e^{{\beta_{0} + \beta_{1} x_{1} + \beta_{2} x_{2} + \cdots + \beta_{p} x_{p} }} }}.$$

Like the gamma regression, the maximum likelihood estimation method was used to estimate the model parameters in the logistic regression model. All the above analyses were implemented in R^59^.

## Data Availability

All the data analyzed in the present study as well as the R script used for the analysis are available online at https://figshare.com/s/571736de2f84f4be615e.
